# Plug-based bail-out strategy for leaflet tear after mitral transcatheter edge-to-edge repair: a case report

**DOI:** 10.1093/ehjcr/ytag218

**Published:** 2026-03-26

**Authors:** Naoki Nishiura, Shunsuke Kubo, Kohei Osakada, Takeshi Maruo, Mitsuru Abe

**Affiliations:** Department of Cardiovascular Medicine, Kurashiki Central Hospital, 1-1-1 miwa, Kurashiki, Okayama 710-8602, Japan; Department of Cardiovascular Medicine, Kurashiki Central Hospital, 1-1-1 miwa, Kurashiki, Okayama 710-8602, Japan; Department of Cardiovascular Medicine, Kurashiki Central Hospital, 1-1-1 miwa, Kurashiki, Okayama 710-8602, Japan; Department of Cardiovascular Medicine, Kurashiki Central Hospital, 1-1-1 miwa, Kurashiki, Okayama 710-8602, Japan; Department of Cardiovascular Medicine, Kurashiki Central Hospital, 1-1-1 miwa, Kurashiki, Okayama 710-8602, Japan

**Keywords:** mitral regurgitation, MitraClip, Valvular heart disease, Complication, Case report

## Abstract

**Background:**

Mitral transcatheter edge-to-edge repair (TEER) is a safe and widespread treatment approach for severe mitral regurgitation (MR). However, rare but serious device-related complications, including single leaflet device attachment, perforation, and leaflet tear, may occur, often requiring surgical correction.

**Case summary:**

An 82-year-old man with congestive heart failure and ischaemic cardiomyopathy underwent mitral TEER using MitraClip XTW clip for severe functional MR. Two days after the procedure, MR recurrence due to a leaflet tear was observed. Given the patient’s advanced age and reduced left ventricular function, our cardiac team decided to perform a transcatheter bail-out procedure. First, the tear was converted to a hole shape by implanting a PASCAL Ace device (Edwards Lifesciences) lateral to the XTW, as this device has a lower risk of leaflet damage compared to the MitraClip device. Then, Amplatzer Vascular Plug II (AVP II) was deployed into the hole, with its mid-lobe positioned in contact with the devices and leaflets. After releasing the AVP II, the MR jet reduced to mild.

**Conclusion:**

An optimal bail-out strategy for leaflet tears has not yet been established. When it is difficult to sufficiently cover the leaflet tear using only an additional TEER device, the use of a plug with additional TEER devices can be considered a bail-out strategy.

Learning points

- No optimal bailout strategy for leaflet tear after TEER has yet been established.
- The use of a plug with additional TEER devices can be considered a bail-out strategy for leaflet tear.

## Introduction

Mitral transcatheter edge-to-edge repair (TEER) using the MitraClip system (Abbott Vascular, Santa Clara, CA, USA) is a safe and widespread treatment approach for severe mitral regurgitation (MR). However, rare but serious device-related complications, including single leaflet device attachment, perforation, and leaflet tear, may occur, often requiring surgical correction.

## Summary figure

**Figure:**
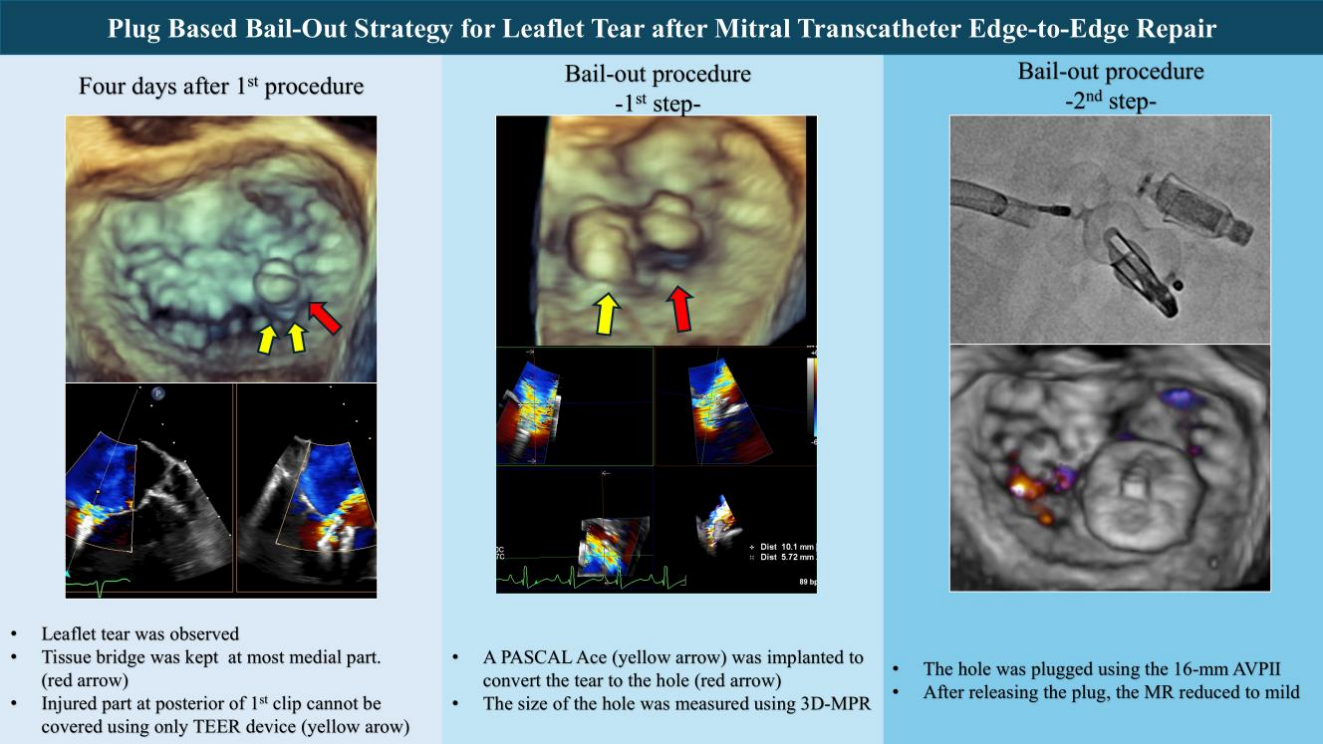


## Timeline

Fourteen days before the bail-out procedure: The patient underwent the first mitral TEER for ventricular functional MR using a MitraClip system.

Twelve days before the bail-out procedure: Transthoracic echocardiography (TTE) revealed MR recurrence due to mitral leaflet tear.

Bail-out procedure: Performed using the PASCAL system and Amplatzer Vascular Plug II.

Two days later: TTE showed mild MR.

Two weeks later: The patient was discharged to home with a good prognosis.

Two months later: No MR recurrence or haemolytic anaemia was noted.

Five months later: The transmitral flow mean gradient gradually increased, which made controlling the heart failure symptom difficult. Consequently, he underwent mitral valve replacement.

## Case presentation

**Figure 1:**
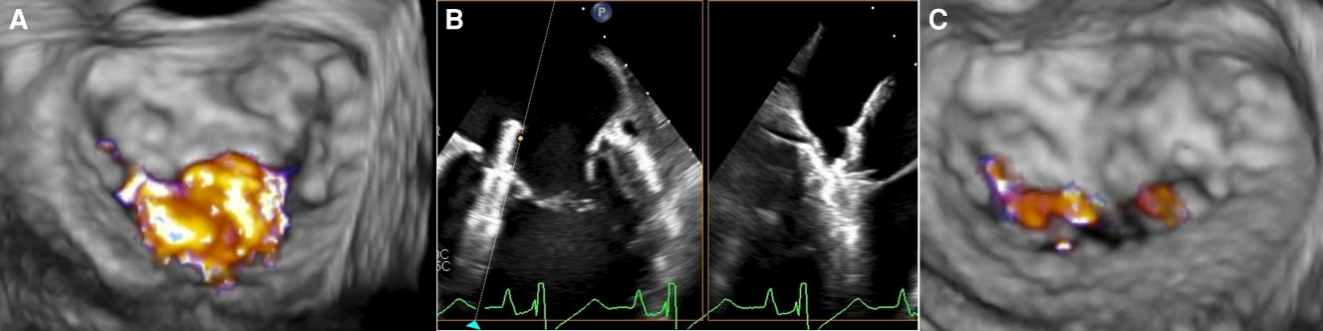
TEE images before and after the first procedure. Baseline 3D-TEE showing severe MR (*A*). XTW implantation for medial A2/P2 (*B*) and mild MR seen in postprocedural TEE (*C*). MR, mitral regurgitation; TEE, transoesophageal echocardiography.

**Figure 2:**
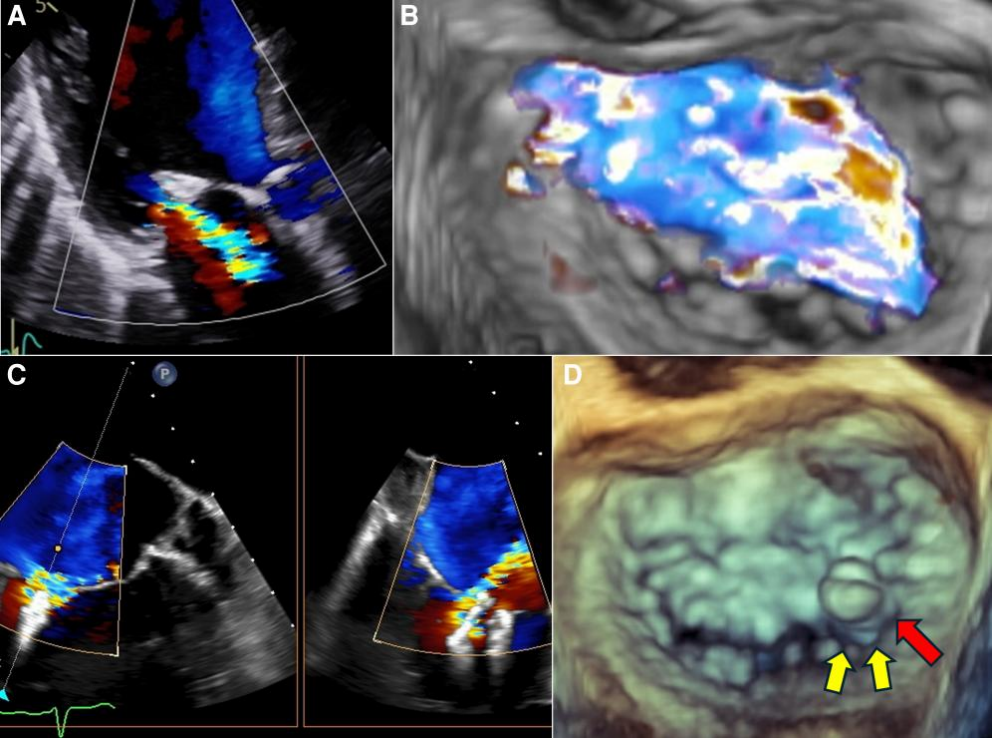
TTE and TEE images after the first procedure. Recurrent severe MR on TTE at 2 days post- transcatheter edge-to-edge repair (*A*). TEE confirming a leaflet tear and an MR jet posterior to the XTW clip (B–C). 3D-TEE showing a torn leaflet posterior to the XTW clip (yellow arrow) and a residual tissue bridge at the clip’s most medial part (red arrow) (*D*). MR, mitral regurgitation; TEE, transesophageal echocardiography; TTE, transthoracic echocardiography.

**Figure 3:**
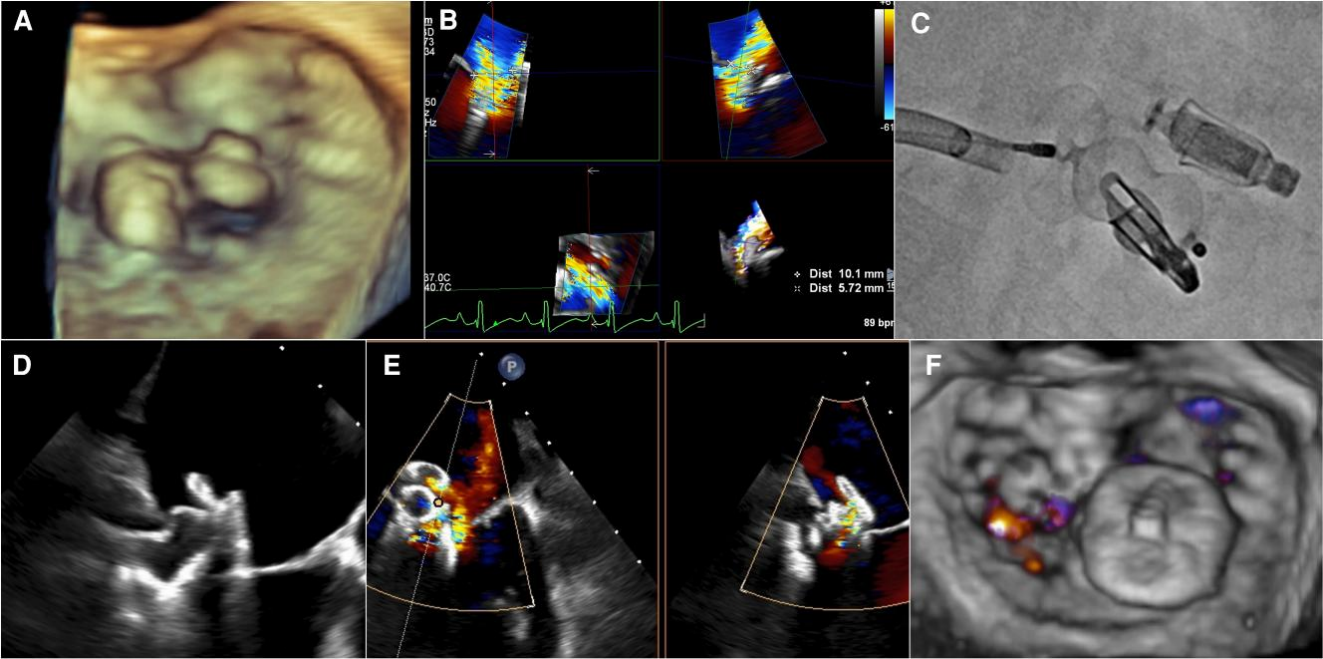
TEE and fluoroscopic images during the bail-out procedure. A hole was created by implanting the PASCAL ACE device (*A*). Based on the 3D multiplanar reconstruction image measurement (*B*), a 16-mm Amplatzer Vascular Plug II device was deployed between the devices (C–D). TEE showing only mild MR postprocedure (E–F). MR, mitral regurgitation; TEE, transoesophageal echocardiography.

An 82-year-old man with congestive heart failure and ischaemic cardiomyopathy complained of dyspnoea on exertion. He had a history of ST-segment elevation myocardial infarction and had undergone percutaneous coronary intervention of the left anterior descending artery 10 years earlier. He had been treated with angiotensin-converting enzyme inhibitor, beta blocker, mineralocorticoid receptor antagonist, and sodium–glucose cotransporter 2 (SGLT2) inhibitor for 1 year prior to the procedure. However, as titrating the mediations was difficult due to hypotension, he remained on diuretics and an SGLT2 inhibitor only. Laboratory findings revealed an elevated N-terminal pro-brain natriuretic peptide level (3406 pg/mL) and chronic kidney disease with an estimated glomerular filtration rate of 45.3 mL/min/1.73 m2. TTE showed a left ventricular ejection fraction (LVEF) of 30% and functional severe MR caused by severe leaflet tethering, consistent with Carpentier type IIIb. Pharmacologic stress myocardial perfusion imaging showed no significant ischaemia. The Society of Thoracic Surgeons predicted the risk of mortality score for mitral valve replacement was 9.82%. Considering his high surgical risk, he underwent mitral TEER for severe ventricular functional MR (*Figure 1A*). A MitraClip XTW clip (Abbott Vascular) was deployed at the medial A2–P2 segment, which improved the MR to mild (*Figure 1B* and *C*). Two days after the first procedure, his heart rate was 78 beats/min; blood pressure, 96/47 mmHg; respiratory rate, 20 breaths/min; and SpO_2_, 96% under ambient air. TTE revealed an LVEF of 23% and severe MR probably due to the leaflet injury (*Figure 2A*). The stroke volume reduced to 25 mL (stroke volume index, 16 mL/m^2^), suggesting a low cardiac output. Chest X-ray showed pulmonary congestion. Transoesophageal echocardiography at 5 days post-procedure showed MR originating just posterior to the clip, indicating a posterior leaflet torn by the clip edge (*Figure 2B* and *C*); however, the tissue bridge remained intact at the clip’s most medial part (*Figure 2D*). Given the patient’s advanced age and reduced LVEF, our cardiac team performed a transcatheter bail-out procedure. Haemodynamic stability and congestion were managed with continuous intravenous dobutamine infusion until the bail-out procedure. Following right femoral venous access, the atrial septum was crossed through the iatrogenic atrial septal defect created during the previous procedure. First, the tear was converted to a hole shape by implanting a PASCAL Ace device (Edwards Lifesciences) lateral to the XTW, as the nitinol device with a passive closing mechanism might have a lower risk of leaflet damage compared to the MitraClip device (*Figure 3A*).^1^ After releasing the PASCAL Ace device, MR jet was observed from a hole just posterior to the XTW clip. Then, the hole was occluded with a 16-mm Amplatzer Vascular Plug II (AVP II, Abbott Vascular), guided by three-dimensional measurements (3D-MPR) (*Figure 3B*). We crossed the hole using a 0.035-inch guide wire with an Agilis steerable sheath (Abbott Vascular), and a 6-Fr multipurpose guiding catheter was inserted into the LV. Subsequently, AVP II was deployed into the hole, with its mid-lobe positioned in contact with the devices and leaflets (*Figure 3C* and *D*, Supplementary material online, *Video S1*). After releasing the AVP II, the MR jet reduced to mild (*Figure 3E* and *F*). The final transmitral mean pressure gradient was 3 mmHg. After the bail-out procedure, the heart failure symptoms were controlled and intravenous dobutamine infusion was tapered and discontinued. The patient was ultimately discharged to home 2 weeks post-procedure. At the 2-month follow-up, mild MR was sustained, and no haemolysis developed. However, the transmitral mean pressure gradient gradually increased to 5 mmHg, which could not be excluded as a contributing factor to the difficulty in controlling heart failure symptoms. Finally, he received mitral valve replacement at another institution at 5 months post-procedure and ultimately died of intestinal ischaemia 2 weeks after the mitral valve replacement.

## Discussion

An optimal bail-out strategy for leaflet tears has not yet been established. Several case reports reported using an additional mitral TEER device to cover a leaflet tear or perforation, provided that the injured site can be adequately covered.^2,3^ However, the indication for this approach depends on the morphology, leaflet injury location, and mitral valve area. In our case, because the leaflet tear was located posterior to the XTW clip, the injured part could not be sufficiently covered by an additional TEER device only. In previous reports, transcatheter plugging was effective in controlling residual MR or leaflet perforation.^4,5^ Our case suggests that using a plug with additional TEER devices may be a viable bail-out strategy for leaflet tears. For this strategy, confirming the residual tissue bridge between first device and injured leaflet is very important. Therefore, the morphology of mitral valve apparatus should be assessed carefully using 3D-MPR. The plug-based bail-out strategy carries risks of haemolysis or mitral stenosis post procedure or worsening leaflet injury during plug deployment. Deploying multiple devices increases the risk of mitral stenosis. Therefore, careful assessment of the mitral valve area, leaflet morphology, and potential bail-out strategies is essential. To minimize these risks, the use of an additional edge-to-edge repair device as a bail-out should be prioritized whenever feasible. In cases where a plug-based bail-out is required, this approach may, in selected patients, serve as a bridging therapy or a palliative treatment.

In summary, we suggest the following bail-out strategies for leaflet tears: (i) If open-heart surgery can be tolerated, bail-out surgical correction should be the first choice; (ii) if the injured area can be grasped and covered using a TEER device, using an additional TEER device alone is recommended; and (iii) when coverage using only an additional TEER device is not feasible, combining an additional TEER device with a vascular plug may be considered; in selected cases, this approach may serve as either a bridging therapy or a palliative treatment for patients at very high surgical risk.

## Supplementary Material

ytag218_Supplementary_Data

## Data Availability

The data underlying this article are available in the article and its online Supplementary material. Further inquiries can be shared on reasonable request to the corresponding author.

## Lead author biography

The lead author graduated from Kobe University in 2017 and currently works as a cardiologist at Kurashiki Central Hospital, Japan. His areas of interest include echocardiography and valvular disease.

## Notes

**Consent:** The patient signed the informed consent form on 8 May 2025 for the use of his health-related data and biological material for research. Written consent for submission and publication of this case report, including images and associated text, was also obtained from the patient in accordance with COPE guidance.
